# Abacus Training Affects Math and Task Switching Abilities and Modulates Their Relationships in Chinese Children

**DOI:** 10.1371/journal.pone.0139930

**Published:** 2015-10-07

**Authors:** Chunjie Wang, Fengji Geng, Yuan Yao, Jian Weng, Yuzheng Hu, Feiyan Chen

**Affiliations:** 1 Bio-X Laboratory, Department of Physics, Zhejiang University, Hangzhou, China; 2 Department of Psychology, University of Maryland, College Park, MD, United States of America; Zhejiang Key Laborotory for Research in Assesment of Cognitive Impairments, CHINA

## Abstract

Our previous work demonstrated that abacus-based mental calculation (AMC), a traditional Chinese calculation method, could help children improve their math abilities (e.g. basic arithmetical ability) and executive function (e.g. working memory). This study further examined the effects of long-term AMC training on math ability in visual-spatial domain and the task switching component of executive function. More importantly, this study investigated whether AMC training modulated the relationship between math abilities and task switching. The participants were seventy 7-year-old children who were randomly assigned into AMC and control groups at primary school entry. Children in AMC group received 2-hour AMC training every week since primary school entry. On the contrary, children in the control group had never received any AMC training. Math and task switching abilities were measured one year and three years respectively after AMC training began. The results showed that AMC children performed better than their peers on math abilities in arithmetical and visual-spatial domains. In addition, AMC group responded faster than control group in the switching task, while no group difference was found in switch cost. Most interestingly, group difference was present in the relationships between math abilities and switch cost. These results implied the effect of AMC training on math abilities as well as its relationship with executive function.

## Introduction

Previous studies have indicated that math abilities play an important role in one’s academic success [1], career aspirations [2, 3], judgment and decision making [4], market and non- market outcomes [4–6], etc. Individual difference in early math abilities is especially important because of its effect on later math development [7, 8]. Therefore, a majority of studies have been dedicated to investigating the potential factors accounting for children’s math abilities [9–11]. One of the most investigated factors is executive function (EF) [12, 13], which commonly refers to higher-order cognitive processes necessary for goal-directed behavior. Based on the “unity and diversity” view, the EF construct contains three separate but related components: working memory, inhibition, and task switching [14]. However, recent studies [15, 16] have further proposed that inhibition is not an independent component of the EF construct, which may be composed only of working memory and task switching.

Working memory refers to a limited capacity involved in storage, manipulation and retrieval of relevant information [17]. It is assumed that problem-solving processes in arithmetic, algebra and geometry often involve the storage of relevant information and retrieval of partial results, which may rely on working memory resources [18]. Task switching refers to the ability switching back and forth between multiple and conflicting conceptual representations, strategies, and/or mental sets [14, 19]. It may also restrain math abilities because solving math problems requires individuals to switch flexibly between different operations, strategies and problem-solving steps [18]. Past research has showed that working memory is a stable predictor of different math abilities [20, 21], but the findings for the associations between task switching and math abilities are still scarce and inconclusive. For example, several studies have shown that task switching is significantly correlated with math abilities [12, 18, 22], while other studies failed to duplicate such relationship [23, 24]. Thus, further exploring the associations between task switching and math abilities is of great importance, which can help us gain a more comprehensive understanding of the underlying cognitive basis for math success and thus develop more effective math instruction methods.

Some studies have revealed that proper interventions can simultaneously improve one’s math abilities and EF [25–27], in line with the finding of a strong relationship between them. Here, we focused on abacus-based mental calculation (AMC) training, a traditional method widely used in Asian countries to help children perform calculations [28]. Initially, Abacus users learn to calculate with a physical abacus, gradually they can get rid of the physical abacus, and calculate extraordinary large numbers via an imagined abacus in their minds with unusual speed. Researchers have demonstrated that AMC training is an effective intervention to improve children’s mental arithmetical ability [28–30], but it remains unclear whether the training affects high-order math abilities. Neuroimaging studies have indicated that abacus experts perform mental calculation by using motor and visual-spatial resources [31, 32] and that AMC training enhances white matter tracts integrity related to visual-spatial processing [33]. Therefore it is reasonable to hypothesize that AMC training might help children improve math abilities in visual-spatial domain. Recent studies have also shown that AMC children have advantages in some other cognitive abilities, such as numerical processing efficiency [34], memory spans [33], simple working memory [35], and general intelligence [36]. In contrast to abundant findings of positive AMC training effects, no study, to our knowledge, has ever reported any detriment effect of AMC training although one study has indicated no differences between AMC experts and controls on the more active aspect of working memory [37]. Moreover, no study to date has assessed whether AMC training affects the task switching aspect of EF. As long-term AMC training enables users to operate beads in different spatial locations within a very short timing window, it may enhance the efficiency switching between complicated contexts.

Furthermore, another intriguing question deserves exploring. Given that both task switching ability and AMC training can enhance individual’s math abilities [22, 28–30, 38], it is possible that an interaction exists between task switching and AMC training in predicting math abilities. Our previous neuroimaging study [32] has indicated that AMC children mainly activated areas of frontal-temporal circuit during simple serial calculation but activated frontal-parietal circuit during complex serial calculation. In contrast, for control children, the activated areas were almost similar during both simple and complex calculation. This implied that when solving math problems with increasing difficulty, AMC children tended to flexibly switch to more appropriate strategies while controls continued with the old strategy. Hence, AMC children might utilize more powers of their task switching ability in solving math problems. Therefore, we hypothesized that the relationship between task switching and math abilities might be stronger in AMC children. That is, AMC training would serve as a moderator in such relationship.

In summary, the current study attempted to explore the impact of long-term AMC training on math abilities, task switching ability and their relationships. Our first prediction was that long-term AMC training would be associated with better performance in both arithmetical and visual-spatial math abilities. Secondly, we predicted that long-term AMC training would also affect the task switching aspect of executive function. Finally, we predicted that long-term AMC training would modulate the relationship between task switching and math abilities (arithmetical ability and visual-spatial ability).

## Materials and Methods

### Ethics Statement

The study was approved by the Institutional Review Board of Zhejiang University and was conducted in accordance with the guidelines of Helsinki Declaration. Written informed consent was obtained from all participants or their guardians before the implementation of the experiments.

### Participants and Procedure

Eighty-two children were recruited from a primary school at school entry and were randomly assigned to either AMC or control groups. All subjects were reported to have no hearing loss, normal or corrected-to-normal visual acuity, no history of neurological disorders and no experience of abacus practice by their parents. We informed all the participants that the study was designed to investigate early child development throughout the whole primary school. Additionally, for participants in the AMC group, we informed them that the study also investigated the developmental effect of AMC training. Thus, we disclosed enough information to the participants and their guardians in order to help them decide if they want to attend our study. Twelve children (10 AMC and 2 control children) were excluded from the study because of their absence in some evaluations and/or school transferring. Hence the AMC group and the control group consisted of 31 (17 boys) and 39 (18 boys) children, respectively.

The baseline evaluation included two questionnaires, a Raven Test and a go/no-go task to measure preschool behaviors, intelligence and inhibition. It was conducted at the start of grade 1 (AMC group: mean age = 6.89 ± 0.41; control group: mean age = 6.90 ± 0.45). Then the AMC group received intensive AMC training for two hours per week at school, while the control children received no physical or mental abacus instruction at or after school. The project was designed to guarantee that both groups had studied the same school curriculum except AMC training. The second and third evaluations were conducted at grade 2 (AMC training length = 9 months; AMC group: mean age = 8.14 ± 0.41; control group: mean age = 8.15 ± 0.45) and at grade 4 (AMC training length = 26 months; AMC group: mean age = 10.22 ± 0.41; control group: mean age = 10.23 ± 0.45) respectively. Both switching task and math test were administrated in these two testing phases. Each evaluation phase lasted approximately two weeks. The go/no-go task and the switching task were tested individually in a quiet room at school, whereas the raven and math tests were administrated in a group.

### Materials

#### Parent Report Questionnaires

The Chinese version of Early School Behavior Rating Scale–Parent Form (ESBS-P) [39, 40] was used to assess parents’ perception of their children’s preschool behaviors in three dimensions: social competence (16 items), anxiety (18 items), and conduct (9 items) problems. All the items were rated by parents on a 4-point rating scale from 1 = hardly ever to 4 = almost always. Besides, the Chinese version of Dimension of Mastery Questionnaire–Parent Form (DMQ-P) [41, 42] was used to assess parents’ perception of their children’s motivation behaviors in six dimensions: object-oriented persistence (9 items), social persistence with adults (6 items), social persistence with children (6 items), gross motor persistence (8 items), mastery pleasure (6 items), negative reaction to failure (5 items). All the items were rated by parents on a 5-point rating scale from 1 = not at all typical to 5 = very typical.

#### Raven Test

The Chinese version of Combined Raven Test for Children [43] was a paper-pencil test. Participants had 40 minutes to complete 6 units (with 12 items in each unit). Each item consisted of a series of geometric figures with one of them missing. Children were asked to find a missed figure among several options. Intelligence raw score was computed for each child and then standardized according to the age norm [44].

#### Go/No-go Task

In the go/no-go task, participants were instructed to respond when they saw animal pictures (go trials) but inhibit their response when they saw chimpanzees (no-go trials). Each animal picture was displayed for 500 ms with an interval of between 1100–1200 ms. There were a practice block of 12 trials and two formal blocks of 70 trials. No-go trials accounted for 20% of the formal trials.

#### Math Test

The Heidelberg Rechentest (HRT) [45] is a standardized math test for primary school pupils in Germany and consists of two subscales evaluating different mathematical abilities. The Chinese version of HRT (CHRT) has been reported to have good reliability (Cronbach’s alphas were above 0.70 for each subscale) and validity (correlation coefficients between each subtest and corresponding subscale ranged from 0.60 to 0.90) [46, 47]. Therefore, the CHRT was used in the current study to measure math abilities.

The subscale of arithmetical ability consisted of six timed subtests: mental addition (e.g. 17 + 15 = _), mental subtraction (e.g. 50–14 = _), mental multiplication (e.g. 15–4 = _), mental division (e.g. 28 ÷ 4 = _), number equations filling (e.g. 4 + _ = 3 + 7) and number comparison (e.g. 2 + 9 _ 20). Trials in each subtest were presented serially with an order of increasing difficulty. Mental multiplication and division were not administered in grade 2 because the second graders had not yet received relevant courses at school.

The subscale of visual-spatial ability included five timed subtests. In the subtest of line estimation, children were required to estimate the length of a series of two-dimensional black lines by comparing them with three presented one-dimensional lines representing length of 1, 5 and 10. In the subtest of pictures counting, children were required to count how many small pictures in each frame. In the subtest of cubes counting, children were required to find out how many small cubes build a three-dimensional figure. In order to give a correct answer, children needed to find out not only the visible cubes, but also the invisible cubes that were completely or partly covered and were necessary to build the figure. In the subtest of number connecting, twenty numbers were randomly presented in each frame and children were required to visually search and connect them with an increasing order of 1 to 20. In the subtest of number sequences, children were required to write three missing numbers in number sequences by deductive reasoning the rule. An additional subtest of copying numbers was first administrated to help children adapt to the other subtests. T scores were computed for these two subscales according to the Chinese adapted city norm in grade 2 and grade 4, respectively.

#### Dots Task

**Fig 1:**
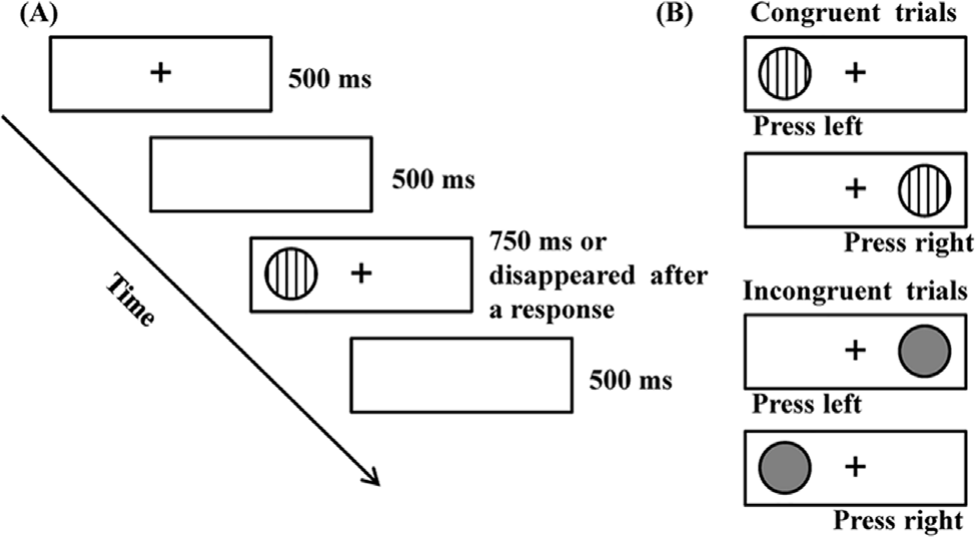
Stimulus and procedure in the Dots task. (A) The time procedure in each trial for the task. (B) Two types of dots were used in the task.

The adapted Dots task [48] was used to measure task switching ability. For each trial, a fixation was presented on the screen for 500 ms with a blank interval followed by for 500ms. Subsequently, the target (either striped or gray dot with diameter equal to 1 cm) was displayed either at the left or right side of the screen up to 750 ms, and disappeared once a response was detected (Fig 1). A blank interval was presented for 500 ms before the onset of next trial. The task consisted of three blocks. The congruent block included 20 trials in which only one type of the dots was presented, and children were asked to press “f” or “j” button on the computer keyboard according to the rule “press on the same side as the dot”. The incongruent block included 20 trials in which another type of the dots was presented, and children were required to inhibit the previously learned rule and press the button on the opposite side of the dot. The mixed block consisted of 61 trials where the above two types of dots were presented. Children were required to remember the rules (e.g., striped same side; gray opposite side) and switch between the two rules. Participants performed four practice trials before entering the formal testing of each block. Half participants in each group were asked to respond in the same side to striped dots and respond in the opposite side to gray dots, while the opposite rule was given to the other half of subjects.

### Data Analysis and Statistics

All analyses were conducted using SPSS 20.0 for windows. In the go/no-go task, inhibitory ability was measured by error rate of no-go trials, accuracy and median RT of go trials. Math abilities were measured by arithmetical and visual-spatial subscales in the CHRT. Two children in control group were excluded from final analysis because their visual-spatial math scores were 3 SD beyond the mean value. In the Dots task, trials with RT faster than 200 ms were excluded from the analyses to prevent anticipation responses [48]. Then accuracy and median RT for correct responses were calculated for each condition. According to Davidson’s study, non-switch trials were defined as the present trials with same rules as the previous trials in the mixed block, while switch trials were defined as the present trials with different rules compared to the previous trials in the mixed block. Local switch cost [19, 48, 49] was then calculated by subtracting accuracy/ median RT in the switch trials from that in the non-switch trials. The sizes of these switch costs were regarded as a measure of task switching ability in the current study (the smaller the switch cost, the better the task switching ability).

## Results

### Baseline Performance

No significant differences between the AMC and control groups were found at baseline evaluation in terms of children’s mean age, gender distribution, preschool behaviors, intelligence, and inhibitory ability, as reported in Table 1.

**Table 1:** Baseline comparisons for the two groups.

		AMC group	Control group	Group differences
Age		6.89(0.41)	6.90(0.45)	*t* (66) = .06, *p* = .95
Gender	percentage of boys	54.84%	43.24%	X ^2^ = .91, *p* = .34
ESBS-P	competence	43.77(5.28)	43.32(8.41)	*t* (66) = .26, *p* = .80
	anxiety	31.23(5.04)	29.78(4.88)	*t* (66) = 1.20, *p* = .24
	conduct	13.90(2.57)	14.89(2.85)	*t* (66) = 1.49, *p* = .14
DMQ-P	object-oriented persistence	3.42(0.49)	3.31(0.59)	*t* (66) = .79, *p* = .43
	social persistence with adults	3.94(0.62)	4.09(0.62)	*t* (66) = 1.03, *p* = .31
	social persistence with children	4.04(0.74)	4.23(0.50)	*t* (66) = 1.27, *p* = .21
	gross motor persistence	3.69(0.61)	3.72(0.56)	*t* (66) = .20, *p* = .84
	mastery pleasure	4.01(0.50)	4.03(0.55)	*t* (66) = .19, *p* = .85
	negative reaction to failure	3.29(0.72)	3.50(0.48)	*t* (66) = 1.41, *p* = .16
	general competence	3.56(0.63)	3.51(0.64)	*t* (66) = .33, *p* = .75
Intelligence		104.52(10.65)	102.92(11.97)	*t* (66) = .58, *p* = .57
Inhibition	accuracy of go trials	0.94(0.04)	0.94(0.04)	*t* (66) = .31, *p* = .76
	error of no-go trials	0.31(0.13)	0.35(0.17)	t (66) = 1.24, *p* = .22
	RT of go trials	549(67)	541(84)	t (66) = 0.46, *p* = .65

### Math Abilities

For children’s performance on both arithmetical and visual-spatial subscales, repeated ANOVA model included Group (AMC or control) as a between-subject factor, and Grade (grade 2 or grade 4) as a within-subject factor. There were main effects of group in both subscales (*F* (1, 66) = 38.26, *p* < .001, *partial η* ^*2*^ = .37; *F* (1, 66) = 17.18, *p* < .001, *partial η* ^*2*^ = .21), indicating that AMC children performed better than control children. In addition, there were a main effect of Grade (*F* (1, 66) = 26.81, *p* < .001, *partial η* ^*2*^ = .29) and an interaction between Group and Grade (*F* (1, 66) = 37.48, *p* < .001, *partial η* ^*2*^ = .36) in the arithmetical subscale. Further analyses with Bonferroni correction indicated that AMC children improved significantly from grade 2 to grade 4 (*p* < .001), which was not present in control children (*p* = .49). Table 2 showed the mean of arithmetical and visual-spatial subscales for each group in both grades.

**Table 2:** Performance in math test and Dots task for both groups.

			Grade 2		Grade 4	
			AMC group	Control group	AMC group	Control group
Math	Arithmetical ability		51.97(7.32)	44.99(9.07)	60.68(8.96)	44.26(8.02)
	Visual-spatial ability		56.65(7.58)	49.29(7.71)	57.16(11.22)	48.69(8.48)
Dots task	Congruent	accuracy	0.99(0.02)	0.95(0.06)	0.99(0.03)	0.99(0.02)
		RT	480(84)	521(84)	378(72)	417(73)
	Incongruent	accuracy	0.90(0.09)	0.90(0.08)	0.96(0.04)	0.96(0.06)
		RT	620(112)	637(110)	473(79)	512(78)
	Mixed	accuracy	0.75(0.11)	0.72(0.12)	0.89(0.08)	0.86(0.10)
		RT	745(106)	800(120)	600(78)	648(74)

### Task Switching

In the Dots task, median RTs were not associated with accuracy in both grades (grade 2: *r* = -.03, *p* > .05; grade 4: *r* = -.21, *p* > .05), indicating no speed-accuracy trade-off. Table 2 showed the mean of median RTs and accuracy for the two groups in the Dots task.

#### RTs

For the two pure blocks, repeated ANOVA model included Group (AMC or Control) as a between-subject factor, and Grade (grade 2 or grade 4) as a within-subject factor. Results showed that AMC group had shorter RTs than control group only in the congruent block, *F* (1, 66) = 6.25, *p* < .05, *partial η* ^*2*^ = .09. Children in grade 4 was faster than in grade 2 in both congruent and incongruent blocks (congruent block: *F* (1, 66) = 93.03, *p* < .001, *partial η* ^*2*^ = .59; incongruent block: *F* (1, 66) = 112.06, *p* < .001, *partial η* ^*2*^ = .63). There were no other main effect of group and any interaction.

**Fig 2:**
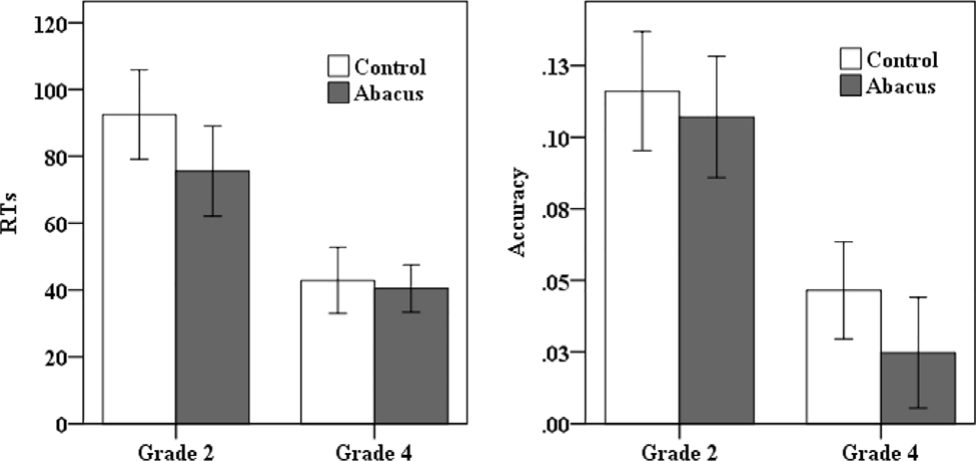
Switch cost measured by mean RT and accuracy for the two groups across grades. The error bars represented one standard error of the mean.

For the mixed block, repeated ANOVA analyses were carried out, including Group (AMC or Control) as a between-subject factor, Switch Type (non-switch or switch) and Grade (grade 2 or grade 4) as within-subject factors. AMC group was faster than control group in RTs, *F* (1, 66) = 7.67, *p* < .01, *partial η* ^*2*^ = .10. RTs in non-switch trials were shorter than in switch trials, suggesting the presence of switch cost on RT, *F* (1, 66) = 134.62, *p* < .001, *partial η* ^*2*^ = .67. Children in grade 4 responded faster than in grade 2, *F* (1, 66) = 91.95, *p* < .001, *partial η* ^*2*^ = .58. There was an interaction between Switch Type and Grade, *F* (1, 66) = 19.69, *p* < .001, *partial η* ^*2*^ = .23. Further analysis indicated that grade 4 had smaller RT switch cost than grade 2 (Fig 2).

#### Accuracy

For the two pure blocks, the 2 (Group) ×2 (Grade) repeated ANOVA model showed that AMC children were more accurate than their peers in the congruent block, *F* (1, 66) = 6.85, *p* < .05, *partial η* ^*2*^ = .09. Grade 4 had higher accuracy than grade 2 (congruent block: *F* (1, 66) = 7.47, *p* < .01, *partial η* ^*2*^ = .10; incongruent block: *F* (1, 66) = 27.38, *p* < .001, *partial η* ^*2*^ = .29). The interaction of Group by Grade was significant in the congruent block, *F* (1, 66) = 7.55, *p* < .01, *partial η* ^*2*^ = .10. Further analysis revealed that AMC children were more accurate than their peers in grade 2, *F* (1, 66) = 8.89, *p* < .01, *partial η* ^*2*^ = .12, which was not found in grade 4.

For the mixed block, the 2 (Group) ×2 (Switch Type) ×2 (Grade) repeated ANOVA model indicated that children were more accurate in non-switch than switch trials, *F* (1, 66) = 53.18, *p* < .001, *partial η* ^*2*^ = .45, suggesting the switch cost on accuracy. Grade 4 had higher accuracy than grade 2, *F* (1, 66) = 93.40, *p* < .001, *partial η* ^*2*^ = .59. Besides, the interaction of condition by grade was also significant, *F* (1, 66) = 15.69, *p* < .001, *partial η* ^*2*^ = .19, suggesting that grade 4 had smaller switch cost measured by accuracy than grade 2 (Fig 2). There were no findings involving group.

### Predicting Math Abilities

To investigate if AMC training modulates the relationship between task switching and math abilities, hierarchical regression was conducted separately for arithmetical ability and visual-spatial ability in both grades. As there were significant correlations between pre-training intelligence and the two math subscales, the intelligence score (standardized score) was entered into the models as covariates in step 1 Group (dummy coded: 0 = control, 1 = AMC) was entered into step 2. Switch cost measured by accuracy/RT (standardized score) was entered in step 3 to determine its incremental predictive validity after controlling for the effects of pre-training intelligence and AMC training. Finally, the interaction term between switch cost and group was entered into step 4. A significant increase in the multiple R^2^ in the last step would indicate moderator effect. In the current study, each switch cost (RT switch cost in grade 2, accuracy switch cost in grade 2, RT switch cost in grade 4 and accuracy switch cost in grade 4) ran a separate analysis in predicting arithmetical ability and visual-spatial ability. The correlation matrix among the study variables were displayed in Table 3.

**Table 3:** Correlations among study variables.

		Baseline	Grade 2				Grade 4		
		Intelligence	RT switch cost	Accuracy switch cost	Arithmetical ability	Visual-spatial ability	RT switch cost	Accuracy switch cost	Arithmetical ability
**Baseline**	Intelligence								
**Grade 2**	RT switch cost	-.34**							
	Accuracy switch cost	-.09	.31**						
	Arithmetical ability	.21	-.06	-.06					
	Visual-spatial ability	.26*	-.07	-.02	.67**				
**Grade 4**	RT switch cost	-.34**	.33**	-.04	-.12	-.23			
	Accuracy switch cost	-.13	.35**	.06	-.13	-.19	.36**		
	Arithmetical ability	.32**	-.25*	-.13*	.74**	.64**	-.32**	-.23	
	Visual-spatial ability	.38**	-.33**	-.08	.56**	.67**	-.31**	-.35**	.72**

Note

* *p* < .05

** *p* < .01.

**Fig 3:**
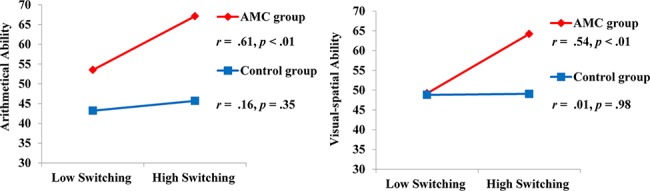
Interaction between AMC training and task switching ability measured by RT switch cost in grade 4 to predict math abilities. The criterion on the y-axis was plotted against two levels of the task switching ability: low switching ability (1 SD above the mean of RT switch cost) and high switching ability (1 SD below the mean of RT switch cost). Plotted regression lines represented the correlations in AMC (red line) and control groups (blue line). Each line represented partial correlations between task switching and math abilities for each group after controlling for pre-training intelligence.

Regression results were expressed in term of R-square change (*ΔR* ^*2*^) accounted by the model and standardized regression coefficients (*β*) of each predictor, which were displayed in Table 4. Our results showed that: (1) in step 1, pre-training intelligence accounted for a significant variance in arithmetical ability in grade 4 and visual-spatial ability in both grades; (2) in step 2, group accounted for an additional significant variance in arithmetical ability and visual-spatial ability for both grades; (3) in step 3, RT switch cost in grade 4 made an additional significant variance in predicting arithmetical ability in grade 4 (model 5, *ΔR* ^*2*^ = .05, *β* = -.24, *p* < .005). Accuracy switch cost in grade 4 provided an additional significant variance in predicting visual-spatial ability (model 8, *ΔR* ^*2*^ = .07, *β* = -.27, *p* < .01); (4) in step 4, the interaction between group and RT switch cost in grade 4 yielded additional significant effects on arithmetical ability (model 5, *ΔR* ^*2*^ = .04, *β* = -.24, *p* < .01) and visual-spatial ability in grade 4 (model 7, *ΔR* ^*2*^ = .09, *β* = -.36, *p* < .005). No outliers (Cook’s distance > 1) were found for all the regression models above, suggesting that these significant findings were not statistical artifact. In order to further examine the nature of the significant interactions, *t* tests of the simple slopes [50, 51] at two levels of moderator (AMC or control) were computed. The results indicated that RT switch cost in grade 4 was negatively related to arithmetical ability (*t* (63) = -3.99, *p* < .01) and visual-spatial ability (*t* (63) = -3.76, *p* < .01) in AMC children, but not in control children (*t* (63) = -1.14, *p* = .26; *t* (63) = -.09, *p* = .93). In addition, Fig 3 showed the relationships between task switching and math abilities in each group.

**Table 4:** Summary of hierarchical regression models using covariates, group, task switching and interactions between group and switch cost as predictors for math abilities.

**Model 1**	**Steps**	**ΔR** ^2^	**Intelligence**	**group**	**RT switch cost** ^**G2**^	**group × RT switch cost** ^**G2**^
Arithmetical ability ^G2^	Step 1	.04	.21			
	Step 2	.14**	.18	.38**		
	Step 3	.00	.20	.38**	.05	
	Step 4	.02	.21	.38**	.18	-.18
**Model 2**	**Steps**	**ΔR** ^**2**^	**Intelligence**	**group**	**Accuracy switch cost** ^**G2**^	**group ×Accuracy switch cost** ^**G2**^
Arithmetical ability ^G2^	Step 3	.00	.18	.38**	-.03	
	Step 4	.01	.16	.38**	.05	-.14
**Model 3**	**Steps**	**ΔR** ^**2**^	**Intelligence**	**group**	**RT switch cost** ^**G2**^	**group × RT switch cost** ^**G2**^
Visual-spatial ability^G2^	Step 1	.07*	.26*			
	Step 2	.18**	.23*	.42**		
	Step 3	.00	.25*	.43**	.06	
	Step 4	.04	.26*	.42**	.22	-.25
**Model 4**	**Steps**	**ΔR** ^**2**^	**Intelligence**	**group**	**Accuracy switch cost** ^**G2**^	**group ×Accuracy switch cost** ^**G2**^
Visual-spatial ability^G2^	Step 3	.00	.23*	.42**	.01	
	Step 4	.01	.21	.42**	.08	-.11
**Model 5**	**Steps**	**ΔR** ^**2**^	**Intelligence**	**group**	**RT switch cost** ^**G4**^	**group × RT switch cost** ^**G4**^
Arithmetical ability^G4^	Step 1	.10**	.32**			
	Step 2	.46**	.27**	.68**		
	Step 3	.05**	.19*	.68**	-.24**	
	Step 4	.04**	.22**	.68**	-.11	-.24**
**Model 6**	**Steps**	**ΔR** ^**2**^	**Intelligence**	**group**	**Accuracy switch cost** ^**G4**^	**group ×Accuracy switch cost** ^**G4**^
Arithmetical ability^G4^	Step 3	.02	.25**	.67**	-.12	
	Step 4	.01	.25**	.67**	-.06	-.10
**Model 7**	**Steps**	**ΔR** ^**2**^	**Intelligence**	**group**	**RT switch cost** ^**G4**^	**group × RT switch cost** ^**G4**^
Visual-spatial ability^G4^	Step 1	.15**	.38*			
	Step 2	.14**	.36**	.38**		
	Step 3	.04	.29*	.38**	-.21	
	Step 4	.09**	.33**	.37**	-.01	-.36**
**Model 8**	**Steps**	**ΔR** ^**2**^	**Intelligence**	**group**	**Accuracy switch cost** ^**G4**^	**group ×Accuracy switch cost** ^**G4**^
Visual-spatial ability^G4^	Step 3	.07*	.32*	.35**	-.27*	
	Step 4	.01	.32*	.35**	-.16	-.16

*Note*: As step 1 and step 2 were the same for model 1 and 2, model 3 and 4, model 5 and 6, model 7 and 8, they were only displayed in model 1, 3, 5 and 7 for the sake of brevity.

* *p* < .05

** *p* < .01.

## Discussion

This study investigated how AMC training affected math abilities and executive functions and modulated their relationships. The results demonstrated that long-term AMC training was associated with better arithmetical and visual-spatial abilities. An interaction between training and switch cost was also found in predicting math abilities, suggesting stronger associations between task switching and math abilities in AMC versus control group.

### Effect of AMC Training on Math Abilities

Consistent with previous studies [28, 30], AMC group was found to perform better in arithmetical subscale than control group, suggesting the effect of AMC training on basic calculation abilities. Importantly, AMC children were also found to outperform their counterparts in visual-spatial subscale. This finding for the first time suggested that AMC training affects math ability in visual-spatial domain, although this training focused on improving calculation speed and accuracy. It is possible that AMC training enabled children to use some strategies that benefit high-order math ability; one of the strategies proposed by previous studies is visual-based strategy [31, 32]. It is speculated that long-term experience of operating physical and/or imagined abacus might promote the construction and formation of mental images, and then improve mathematical performance in visual-spatial domain.

### Effect of AMC Training on Task Switching

AMC children were found to respond faster than their peers in the mixed block requiring switching back and forth between two conflicting rules. The finding was in line with our prediction that AMC training might affect the ability to switch between different mental sets. Previous neuroimaging studies have reported that the frontal-parietal network is involved in both abacus mental calculation [31, 32, 52] and task switching processes [53–55], suggesting that these two processes may share similar underlying neural substrates. It might be the neural mechanism that why AMC training benefited response speed in switch task. Such benefit may also reflect the influence of AMC training on working memory as the switching task used in the current study was suggested to tap the cognitive process of working memory in addition to switching process [49].

Our longitudinal analysis suggested that the switch cost decreased significantly from grade 2 to grade 4, which was consistent with some cross-sectional studies [56, 57]. This study did not reveal any effect of AMC training on switch cost. One explanation was that the absence of AMC training effect was related to immature task switching process. It has been proposed that children development driven by typical age-related development and training-induced plasticity share some common underlying neural mechanisms [58]. Especially, the underlying neural circuits for executive function are still developing until late childhood and adolescence [59]. Therefore, we assumed that such development for children in this study might have buffered the effect of AMC training on switching ability. A previous study supported this argument by finding that only young adults but not children showed training-related benefits [60].

### Effect of AMC Training, Task Switching Ability and Their Interactions on Math Abilities

The most important finding of the current study was that long-term AMC training modulated the relationships of task switching ability to arithmetical and visual-spatial abilities for fourth graders. For AMC children, task switching ability was positively related to math abilities even when early general intelligence was controlled, which, however, was not found in control children. The finding supported our hypothesis that AMC children showed stronger associations between task switching and math abilities. Our recent study has demonstrated that AMC children showed increased functional activations and resting-state functional connectivity within the frontal-parietal network in comparison with control children [60], providing evidence that the AMC training might enhance the efficiency of the frontal-parietal network. Interestingly, this network is involved in both task switching and math abilities [53, 61], thus, the shared underlying neural mechanism may lead to the more significant correlations between math abilities and executive function in AMC versus control group. Such interpretation may be supported by a recent fMRI study [62] showing that the reorganization of functional connectivity within the frontal-parietal network modulated post-training working memory performance.

Besides, intelligence measured before administrating AMC training was found to be related to math abilities later. This finding supported the claim that individual differences in early cognitive ability are related to later math learning [63]. Another strength of the current study was that intelligence was controlled in the relationship between task switching and math abilities, which was reported to be an limitation in a previous meta-analysis [22]. In line with a few studies [64, 65], our study showed that task switching ability is, over and above early intelligence, an important contributor for math abilities.

### Limitations

One important limitation of the current study was that we did not assess children’s baseline math and task switching abilities. The lack of these baseline evaluations could not guarantee that the samples were equivalent in math and task switching before the AMC training started. Thus, group differences in our findings might be driven by pre-training group differences other than AMC training. However, it is ensured that both groups were matched in age, gender, problem and motivation behaviors, general intelligence, and inhibitory ability during the baseline evaluation. Additionally, parents and teachers reported that children in both AMC and control groups had never learned math consistently before first grade. That is to say, the two groups were matched as closely as possible, which might reduce the possibility that the findings in this study were driven by pre-training group differences. Another limitation in the current study was that only one switching task was used because different switching tasks tap the different aspects of executive functions [48]. Therefore, future work should consider stricter baseline control and better study design.

## Conclusions

The current study for the first time suggested that AMC training not only affect high-order math abilities in children aged between 7 and 11 but also modulate the relationships between switch cost and math abilities. The current work expanded our understanding of the relationships between executive functions, interventions, and math achievements, and thus might help develop effective training programs for children at risk for mathematical learning inabilities.

## Supporting Information

S1 Dataset The dataset for the current study. (SAV)
